# Supplementary material for: Integrative Analysis of miR-21, PTEN, and Immune Signatures in Colorectal Cancer
Source: Int J Mol Sci. 2025 Dec 17;26(24):12118. doi: 10.3390/ijms262412118 (PMC12732930; doi:10.3390/ijms262412118)
Supplement: Supplementary file 1 [file ijms-26-12118-s001.zip › ijms-4030882-supplementary.pdf]

Supplementary Materials

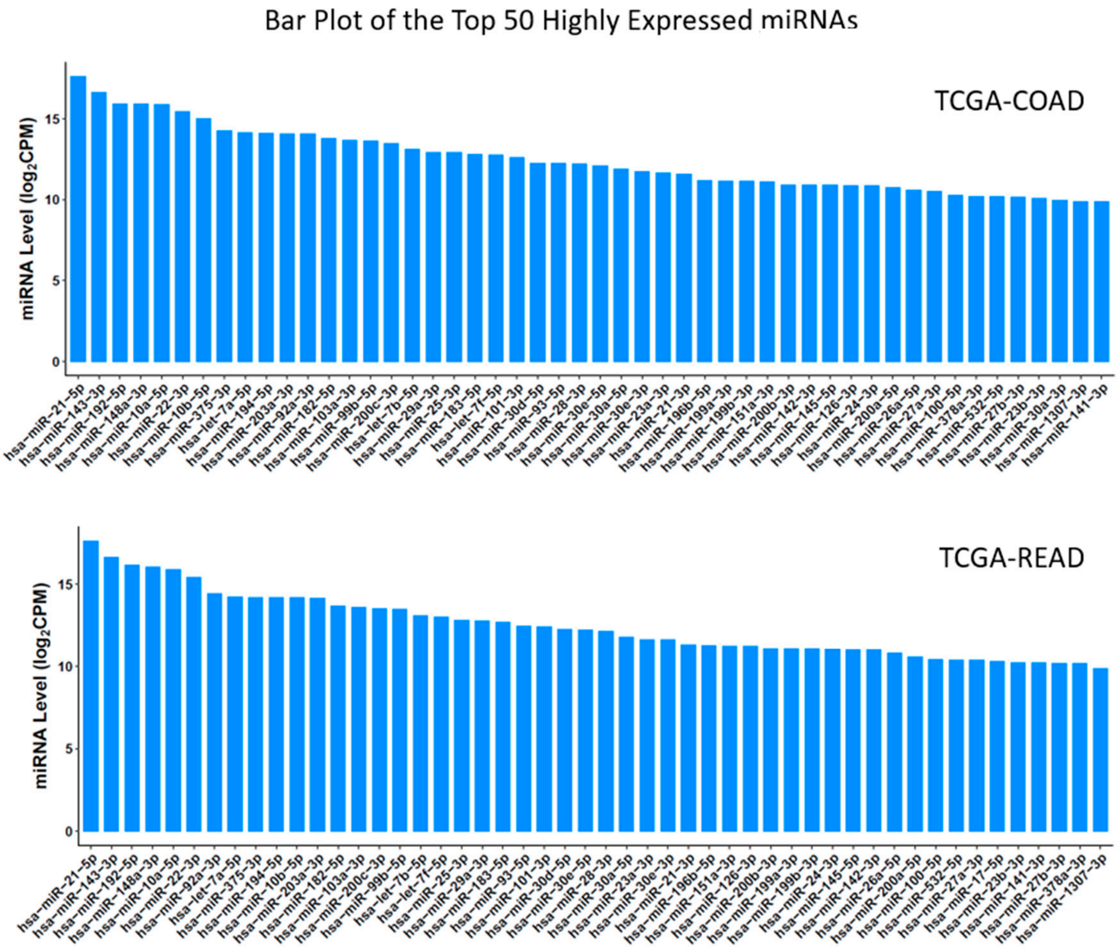

Figure S1. Top 50 most abundant miRNAs in TCGA-COAD and READ. Bar plots displaying the top 50 miRNAs ranked by mean expression (log_2 CPM) in (top) COAD and (bottom) READ samples. X-axis denotes miRNA names; Y-axis shows log_2 counts per million.

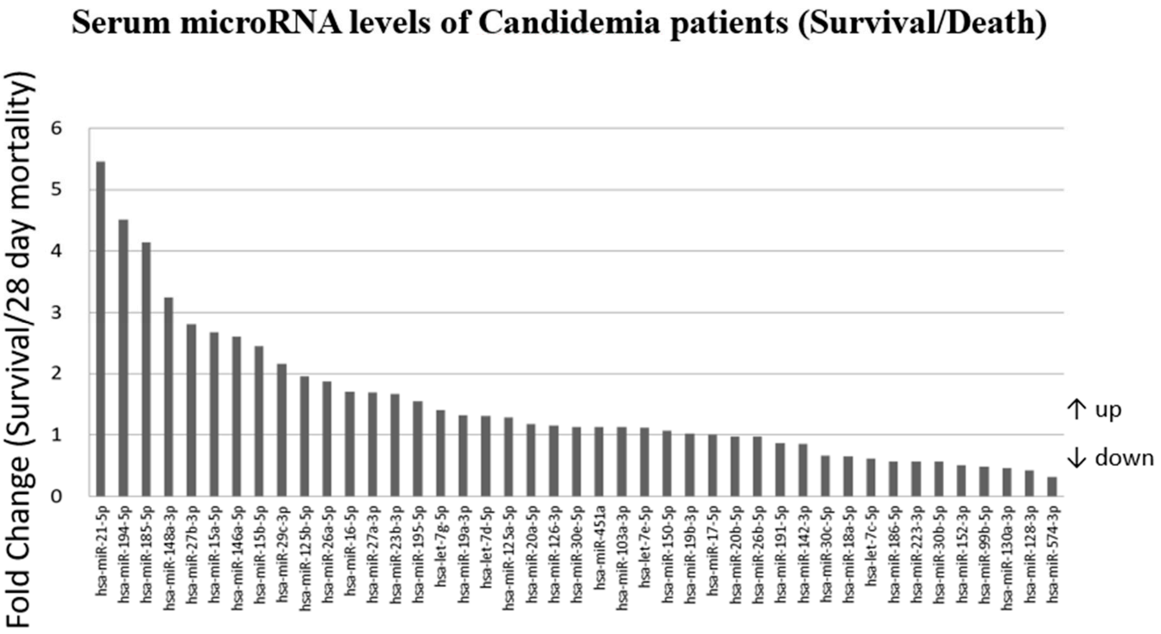

Figure S2. Differential serum miRNA expression in candidemia patients by 28-day survival status. Serum miRNA profiles were analyzed in candidemia patients stratified by 28-day survival. Fold changes in miRNA expression are presented, with upregulated miRNAs (fold change > 1) and downregulated miRNAs (fold change < 1) highlighted. Total RNA was extracted from patient serum and profiled using GeneChip miRNA arrays.
